# Schizophrenia polygenic risk score and 20-year course of illness in psychotic disorders

**DOI:** 10.1038/s41398-019-0612-5

**Published:** 2019-11-14

**Authors:** Katherine G. Jonas, Todd Lencz, Kaiqiao Li, Anil K. Malhotra, Greg Perlman, Laura J. Fochtmann, Evelyn J. Bromet, Roman Kotov

**Affiliations:** 1 Department of Psychiatry, Stony Brook University, New York, NY USA; 2 Departments of Psychiatry and Molecular Medicine, Donald and Barbara Zucker School of Medicine at Hofstra/Northwell, East Garden City, USA; 3 Division of Psychiatry Research, Zucker Hillside Hospital, Northwell Health, New York, NY USA; 4 Center for Psychiatric Neuroscience, Feinstein Institute for Medical Research, New York, NY USA; 5 Department of Applied Mathematics and Statistics, Stony Brook University, New York, NY USA; 6 Department of Pharmacological Sciences, Department of Biomedical Informatics, Stony Brook University School of Medicine, New York, NY USA; 7 Department of Family, Population & Preventive Medicine, Stony Brook University, New York, NY USA

**Keywords:** Clinical genetics, Predictive markers, Diagnostic markers, Schizophrenia

## Abstract

Understanding whether and how the schizophrenia polygenic risk score (SZ PRS) predicts course of illness could improve diagnosis and prognostication in psychotic disorders. We tested whether the SZ PRS predicts symptoms, cognition, illness severity, and diagnostic changes over the 20 years following first admission. The Suffolk County Mental Health Project is an inception cohort study of first-admission patients with psychosis. Patients were assessed six times over 20 years, and 249 provided DNA. Geographically- and demographically-matched never psychotic adults were recruited at year 20, and 205 provided DNA. Symptoms were rated using the Schedule for the Assessment of Positive Symptoms and Schedule for the Assessment of Negative Symptoms. Cognition was evaluated with a comprehensive neuropsychological battery. Illness severity and diagnosis were determined by consensus of study psychiatrists. SZ PRS was significantly higher in first-admission than never psychotic groups. Within the psychosis cohort, the SZ PRS predicted more severe negative symptoms (*β* = 0.21), greater illness severity (*β* = 0.28), and worse cognition (*β* = −0.35), across the follow-up. The SZ PRS was the strongest predictor of diagnostic shifts from affective to non-affective psychosis over the 20 years (AUC = 0.62). The SZ PRS predicts persistent differences in cognition and negative symptoms. The SZ PRS also predicts who among those who appear to have a mood disorder with psychosis at first admission will ultimately be diagnosed with a schizophrenia spectrum disorder. These findings show potential for the SZ PRS to become a tool for diagnosis and treatment planning.

## Introduction

The SZ PRS agglomerates the weighted effect of many single-nucleotide polymorphisms (SNPs) that discriminate schizophrenia cases from healthy controls^1^. As the size of the PRS discovery cohort has increased, observed case–control variance explained has increased from 3 to 18%^1,2^. However, there is substantial heterogeneity in both the clinical presentation and illness course of schizophrenia that is difficult to capture in large studies. Carefully phenotyped, longitudinal studies may reveal when and how the SZ PRS’s effects unfold.

Evidence on the association between SZ PRS and clinical presentation has been inconsistent. For example, a study of adults found an association with mood-incongruent psychosis in both schizophrenia and bipolar samples^3^, suggesting the PRS is especially sensitive to positive symptoms, while a study of an adolescent cohort found that the SZ PRS predicted negative symptoms and anxiety^4^. Still others have found no associations between SZ PRS and symptom dimensions^5,6^. Furthermore, although symptom course is a central component of the diagnostic criteria for schizophrenia, the association between the SZ PRS and symptom trajectories in psychotic disorders is unknown. In comparison with links with symptoms, the association of SZ PRS with neurocognitive deficits has been stronger. The SZ PRS has been linked with prodromal motor deficits^7^, as well as cognitive decline and lower educational attainment in the general population^8,9^. However, it is unclear whether SZ PRS predicts either cognition or cognitive decline in people with psychotic disorders^10^.

Investigating the longitudinal associations of the SZ PRS is important, because diagnosis is often incorrect at first admission. Due to diagnostic error, cross-sectional studies of first-admission patients may underestimate the magnitude of genetic effects, which gain power as the diagnostic picture clarifies^11–13^. At the time of first onset, the SZ PRS distinguishes patients from controls^14^, and predicts treatment response^15^. Later in the course of illness, the SZ PRS distinguishes between schizophrenia, bipolar disorder with psychosis, and bipolar disorder without psychosis^3^. These effect sizes have been modest, and it remains unclear whether the effect of the SZ PRS is large enough to be clinically useful. Investigating the association between the SZ PRS and diagnosis over time may reveal genetic effects that are dampened by inaccurate diagnoses, as well as genetic liabilities for specific diagnostic trajectories.

If the SZ PRS is a robust predictor of diagnostic changes, illness severity over time, trajectory of specific symptoms, or cognitive changes, such a pattern could have significant implications for diagnosis, prognosis, and treatment. Given that outcomes in psychotic disorders differ substantially across patients^16,17^, the SZ PRS may help direct appropriate treatment to those who will need it most. Our primary aim was to determine whether the SZ PRS predicted stable differences in symptoms, or worsening symptom trajectories. Our secondary hypothesis was that the SZ PRS would also predict diagnostic shifts, as psychotic disorder diagnoses are determined by symptom trajectories. This study addresses these hypotheses by investigating the contributions of the SZ PRS to trajectories of symptom and illness severity, cognition, and diagnosis over 20 years.

## Methods

### Sample

Data were drawn from the Suffolk County Mental Health Project, a longitudinal first-admission study of psychosis. Between 1989 and 1995, individuals with first-admission psychosis were recruited from the 12 inpatient facilities in Suffolk County, New York (response rate 72%). The Stony Brook University Committee on Research Involving Human Subjects and the review boards of participating hospitals approved the protocol annually. Written consent was obtained from all study participants or their parents, for those who were minors at baseline. Eligibility criteria included residence in Suffolk County, age between 15 and 60, ability to speak English, IQ > 70, first admission within the past 6 months, current psychosis, and no apparent medical etiology for psychotic symptoms.

A total of 628 participants met inclusion criteria. Follow-up interviews were conducted at 6 months, 24 months, 48 months, 10 years, and 20 years after baseline. Ninety-two participants died during the follow-up period. Of those surviving, 373 were interviewed at the 20-year follow-up^18^. Of those 373, DNA was collected from 249 participants as part of the Genomic Psychiatry Cohort^19^, and is accessible through dbGaP.

At the 20-year point, a comparison group of 261 (205 provided DNA) never psychotic adults was recruited using random digit dialing in zip codes where members of the psychosis cohort resided^20^. Rate of participation in this group was 67%. The comparison group was sex- and age-matched to the psychosis cohort. Table 1 reports demographics for both the psychosis and never psychotic cohorts.

**Table 1:** Demographic characteristics

	Psychosis cohort (*n* = 249)		Never psychotic adults (*n* = 205)	
	*N*	%	*N*	%
*Gender*				
Male	142	57.0	118	57.6
Female	107	43.0	87	42.4
*Race/ethnicity*				
Caucasian	208	83.6	184	89.8
African–American	27	10.8	9	4.4
Other	14	5.6	12	5.8
*Age at 20-year follow-up*				
30–40	54	21.7	32	15.6
40–50	106	42.6	80	39.0
50–60	63	25.3	59	28.8
60–70	24	9.6	33	16.1
>70	2	0.8	1	0.5
*Diagnosis at 20-year follow-up*				
Schizophrenia	99	39.8	−	−
Schizoaffective disorder	34	13.7	−	−
Substance-induced psychosis	12	4.8	−	−
Other psychoses	19	7.6	−	−
Major depression	21	8.4	−	−
Bipolar disorder	64	25.7	−	−
AP medication	142	57.0	4	2.0
*Illness severity (GAF)*				
<20	2	0.8	−	−
21–40	115	46.2	−	−
41–60	70	28.1	31	15.1
61–80	46	18.5	112	54.6
>80	16	6.4	62	30.3

*GAF* global assessment of functioning

For genetic assays, DNA was extracted from peripheral lymphocytes and genotyped using the Illumina PsychArray-8 platform containing 571,054 markers. Standard quality control procedures were performed to exclude SNPs with minor allele frequency (MAF) < 1%, genotyping failure > 5%, Hardy–Weinberg equilibrium *p* < 10^−6^, mismatch between recorded and genotyped sex, as well as related individuals (π̂ > 0.20, in which case the relative with the lower call rate was dropped). Mean call rate was 99.8%. SNP imputation was conducted with IMPUTE2^21^, against the full 1000 Genomes phase 3 reference panel^22^. The imputed SNPs underwent another round of quality control and SNPs with missing data > 5% and imputation information score < 0.8 were excluded, yielding 6.87 M high-quality biallelic SNPs. Genomic data analysis was performed using the SVS software, version 8.7.0^23^.

The PRS was calculated for each participant in the sample as the weighted sum of the risk allele they carried, based on the summary statistics (effect alleles and odds ratios) derived from the clumped PGC-2 GWAS results, which consists of 102,636 SNPs^2^. The clumped PGC GWAS summary statistics file was downloaded from the LD Hub at the Broad Institute (http://ldsc.broadinstitute.org/ldhub/). SNPs were clumped to a more significant SNP if they were in LD (*r*^2 ^≥ 0.10) within a 500 kb window. PRS calculation was carried out in the PRSice software^24^. The SZ PRS was calculated at several *p*-value thresholds (*p* ≤ 5 × 10^−8^, 0.001, 0.01, 0.05, 0.10, 0.20, and 0.50). Mean PRS scores at these thresholds for each diagnostic group are reported in Supplementary Table 1. The results presented here utilize PRS scores based on SNPs with a *p*-value < 0.01, as this threshold provided the greatest separation between the diagnostic and never psychotic groups. However, analyses scores based on other thresholds yielded similar results (available upon request).

### Measures

#### Diagnosis

Research diagnoses were made by the consensus of study psychiatrists at baseline and again at year 20 using all available longitudinal information, including results of the SCID^25^, interviews with participants’ significant others, medical records, and observations and behavioral ratings by masters-level interviewers. Diagnoses were made according to DSM criteria. The diagnostic process is described by Bromet^11^.

#### Symptoms

Symptom domains were rated using the Scale for the Assessment of Positive Symptoms (SAPS;^26^), and the Scale for the Assessment of Negative Symptoms (SANS;^27^). Masters-level mental health professionals made ratings of symptoms based on their interview of the participant, interviews with significant others, and medical records. SAPS and SANS ratings were used to score four factor-analytically derived subscales developed in a prior publication^28^. The subscales were composites of nonoverlapping items with highest loadings on a given factor. Internal consistency of subscales was adequate: reality distortion (delusions/hallucinations; Cronbach’s α = 0.85) and disorganization (α = 0.77) from the SAPS; avolition (α = 0.87) and inexpressivity (α = 0.90) from the SANS^28^. Sample sizes at each time point were as follows: 6 months (*n* = 217); 24 months (*n* = 209); 48 months (*n* = 192); 10 years (*n* = 234); and 20 years (*n* = 246). Depression in the past month was rated via the Structured Clinical Interview for DSM-III-R at baseline^25^ and DSM-IV thereafter^29^, administered without skip-outs and scored as a sum of nine symptom ratings. The Global Assessment of Functioning (GAF) was used to rate overall illness severity (symptoms plus functional impairment) by the consensus of study psychiatrists using all available information.

#### Cognition

The neuropsychological battery at 24-month and 20-year follow-up included Verbal Paired Associates and Visual Reconstruction (WMS-R, immediate and delay trials;^30^), Symbol-Digit Modalities (WAIS-III;^31^), Trails A and B^32^, the Controlled Oral Word Association Test^33^, Vocabulary (WRAT-3;^34^), and the Stroop Test^35^. Sample sizes for the first-admission cohort were *n* = 201 at 24 months and *n* = 224 at 20 years.

### Analyses

Code for all analyses is available from the corresponding author on request.

#### Attrition

Supplemental Table 2 describes the sample sizes and means available for each outcome measure at each time point, as well as contrasts between those who did and did not provide DNA. Those who provided DNA were slightly younger (Cohen’s *d* *=* −0.18, *p* < 0.05), more likely to be prescribed antipsychotic medications at 48 months (Cramer’s V = 0.17, *p* < 0.05), 10 years (Cramer’s V = 0.10, *p* < 0.05), and 20 years (Cohen’s *d* *=* 0.17, *p* < 0.05), had higher ratings on SANS avolition at baseline (Cohen’s *d* = 0.18; *p* < 0.05), and lower ratings at 48 months (*d* *=* −0.23; *p* < 0.05), and had better scores on the COWAT (*d* = 0.60, *p* < 0.05). All analyses used full information maximum likelihood estimation, which uses all data, including partial cases, to arrive at unbiased parameter estimates.

#### Baseline associations

The baseline time point was qualitatively different from follow-up time points, as all participants were selected because they were actively psychotic. For this reason, baseline symptoms and trajectories from 6 months to 20 years were analyzed separately. Associations of the SZ PRS with baseline symptoms and illness severity were tested via linear regression adjusted for age.

#### Trajectories

Multilevel spline regression models were used to estimate trajectories of symptoms and illness severity. To allow for nonlinear trajectories, we identified the point at which the average trajectory changed direction. The placement of the change point was determined by alternatively placing it at each 1-year interval from baseline to 20-year follow-up, and comparing the fit of these competing models via the Bayesian Information Criterion (BIC).

#### Cognitive change

Parallel analysis was used to determine how many factors were reflected in the set of cognitive tests administered at the 24-month and 20-year follow-ups. In both cases, parallel analysis indicated a single cognitive factor. One-factor confirmatory measurement models were fit to the cognitive tests at each time point. In both the 24-month and 20-year models, all test loadings on the general factor were >0.3 and statistically significant. Residual covariance terms were included between subscales of tests with more than one subscale. Model fit was excellent at both 24 months (CFI = 0.94; RMSEA = 0.04) and 20 years (CFI = 1.00; RMSEA = 0.01). The latent cognition factor was regressed on the PRS and age.

#### Diagnosis and diagnostic shifts

Supplemental Table 3 reports Cohen’s *d* and R^2^ of the SZ PRS scores for diagnostic groups at baseline and 20-year follow-up relative to never psychotic adults. *R*^2^ is Lee’s^37^ coefficient of determination on the liability scale, corrected for case–control ascertainment. Baseline and 20-year diagnoses were dichotomized into affective psychosis and non-affective psychosis. The affective psychosis (AP) category included psychotic bipolar disorder and psychotic major depression. The non-affective psychosis (NAP) category included schizophrenia, schizoaffective disorder, substance-induced psychosis, and other psychoses. This categorization was made based on the findings reported as described by Kotov^36^, which showed that patients who experienced 10 or more days of psychosis outside of a mood episode had worse outcomes at 10-year follow-up. All participants with schizophrenia as well as large majority of those with substance-induced psychosis and other psychoses met this criterion, and were therefore included in the NAP group. This grouping was consistent with similarity of PRS scores among specific diagnoses.

Predictive modeling of shifts from AP to NAP between baseline and 20 years was performed by regressing diagnostic shift groups on statistically significant clinical predictors from Table 3 of Bromet^11^. Jackknife resampling and leave-one-out cross-validation were used to calculate the stability of model estimates and prediction error, respectively. Sensitivity analyses were performed, excluding patients with a diagnosis of substance-induced psychosis or other psychoses at either baseline of 20-year follow-up, in order to confirm that results were not driven by these potentially ambiguous cases. Results from these analyses are reported under “Sensitivity Analyses” in Supplementary Material.

#### Population stratification

In order to control for population stratification due to ancestry, all analyses were covaried on the first ten principal components of genetic covariance^38^. Because the PGC-2 SZ PRS was calibrated in largely European ancestry samples, it is less accurate in non-European samples^39^. For this reason, we completed sensitivity analyses on the subsample of participants within three standard deviations of the mean on the first four principal components of genetic covariance (*n* = 235). These results are reported in Supplementary Table 4 and under “Sensitivity Analyses” in Supplementary Material.

#### Multiple comparisons

To limit the number of Type I errors, we employed Benjamini and Hochberg’s procedure for controlling the false-discovery rate at *q* = 0.10^40^. Among the 46 contrasts completed in these analyses, all reported *p*-values remain significant after FDR, with one exception noted in Footnote 2.

## Results

### Baseline associations

The SZ PRS was not associated with severity of hallucinations and delusions, disorganization, or inexpressivity at first admission. There was an effect of the PRS on avolition (*R*^2^ = 0.11, β = 0.33, 95% CI 0.10–0.57, *p* < 0.01). The PRS was also not associated with depression or global illness severity.

### Symptom trajectories

**Fig. 1:**
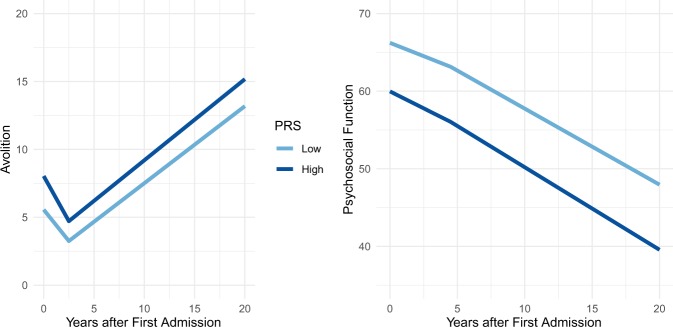
Effect of SZ PRS on course of illness. The course of negative symptoms (SANS avolition, left) and illness severity (GAF, right) over the 20 years following first hospitalization. Light and dark blue lines depict the course of illness for those with low and high SZ PRS scores, respectively, as defined by a median split

Table 2 reports the associations of the SZ PRS with trajectories of symptoms and global illness severity. The SZ PRS was not associated with hallucinations/delusions, disorganization, inexpressivity, or depression. Rather, the SZ PRS was associated with stable differences in avolition (*R*^2^ = 0.04, β = 0.21, 95% CI 0.04–0.39, *p* < 0.05) and illness severity (*R*^2^ = 0.08, GAF; β = −0.28, 95% CI −0.47 to −0.10, *p* < 0.01). The SZ PRS did not predict changes in symptoms over time, but stable differences across all time points. Figure 1 depicts the mean trajectory of illness severity and avolition for those with high versus low PRSs.

**Table 2:** Symptom trajectories over 20 years

	Mean effect of SZ PRS				SZ PRS on course part 1				SZ PRS on course part 2			
	*R*^2^	β	95% CI	*p*	*R*^2^	β	95% CI	*p*	*R*^2^	β	95% CI	*p*
Hallucinations/delusions	<0.01	0.04	[−0.12, 0.20]	0.64	<0.01	0.01	[−0.11, 0.14]	0.86	–	–	–	–
Disorganization	0.01	0.09	[−0.08, 0.26]	0.32	<0.01	0.02	[−0.12, 0.15]	0.82	<0.01	0.03	[−0.12, 0.18]	0.74
Avolition	**0.04**	**0.21**	**[.04, 0.39]**	**0.02**	<0.01	−0.03	[−0.12, 0.07]	0.59	<0.01	−0.01	[−0.12, 0.09]	0.79
Inexpressivity	0.02	0.14	[−0.04, 0.33]	0.13	0.01	−0.07	[−0.18, 0.04]	0.21	<0.01	−0.05	[−0.17, 0.06]	0.34
Depression	0.01	0.12	[−0.04, 0.28]	0.13	0.01	−0.10	[−0.20, 0.00]	0.06	–	–	–	–
Illness severity	**0.08**	**−0.28**	**[−0.47, −0.10]**	**<0.01**	<0.01	−0.03	[−0.12, 0.06]	0.50	<0.01	.04	[−0.05, 0.12]	0.37

*R*^2^ is the coefficient of determination on the liability scale, corrected for case–control ascertainment. The mean effect of the SZ PRS indicates an effect that is constant across all time points. SZ PRS on course is the effect of the SZ PRS on change in symptoms over time. For reality distortion and depression, symptom trajectories are linear, so there is only one effect on slope. Trajectories have inflection points for disorganization (13.5 years after baseline), avolition (4.5 years), inexpressivity (2.5 years), and illness severity (4.5 years), so effect of PRS is also modeled on the second slope for these domains

Bold values are significant at *p* < 0.05

### Cognitive outcomes

The SZ PRS was negatively associated with the latent cognitive factor at 24 months (*R*^2^ = 0.08, β = −0.29, 95% CI −0.04 to −0.55, *p* < 0.05) and at 20 years (*R*^2^ = 0.12, β = −0.35, 95% CI −0.13 to −0.55, *p* < 0.01), though not with change between the two time points. Given the correlation between higher negative symptoms and lower cognitive performance (*r* = −0.32 at 24 months and *r* = −0.44 at 20 years), we covaried the models for avolition, but this did not change the pattern of effects at 20 years^1^.

### Diagnostic shifts

**Fig. 2:**
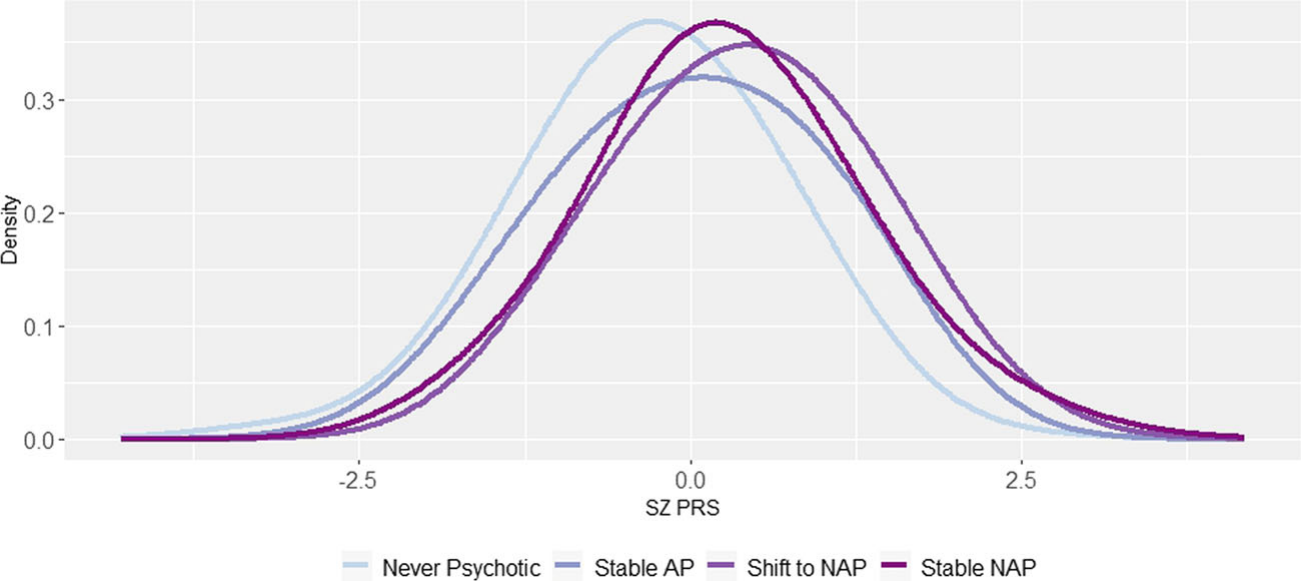
Distribution of SZ PRS scores in diagnostic shift groups. AP is affective psychosis; NAP is non-affective psychosis. Density represents the probability of observing a given PRS score within a given diagnostic shift group. All SZ PRS scores are standardized relative to never psychotic adults

We next tested whether the SZ PRS predicted shifts between affective psychosis (AP) and non-affective psychosis (NAP). Those in the NAP group at both baseline and 20 years had higher SZ PRS scores than those who were in the AP group at both times points (*d* = 0.27, *p* < 0.05). Notably, those whose baseline AP diagnosis was changed to NAP by the 20-year follow-up had larger SZ PRS scores compared with participants whose diagnosis was AP at both time points (*d* = 0.45, *p* < 0.05; equivalent to AUC = 0.62), and were not distinguishable from those who were in the NAP group at both time points (*d* = 0.14, *p* = 0.55). Figure 2 depicts the distribution of SZ PRS scores for these groups. At high levels of the SZ PRS, liability for NAP (either stable or shifted into NAP) versus stable AP was pronounced. At the highest decile, the SZ PRS detected those who will transition into NAP group with 68% accuracy (sensitivity = 16%, specificity = 92%, full results in Supplemental Table 5 and Supplementary Fig. S1).

To compare the performance of the SZ PRS to clinical predictors, we added significant predictors from Bromet^11^—baseline GAF, negative, psychotic, disorganized, depressive, and manic symptoms; antipsychotic and mood stabilizers prescriptions—to the regression described above. In the resulting model, only treatment with mood stabilizers (AUC = 0.63) and the SZ PRS (AUC = 0.61) predicted a shift from AP to NAP (total AUC = 0.70; leave-one-out prediction error = 0.33)^2^.

## Discussion

As the size of the calibration sample for the SZ PRS has increased, so has the power to distinguish cases from controls^14,41^. However, it has remained unclear how the SZ PRS influences psychotic disorders over time. Taken together, these analyses show that the SZ PRS is not only a robust predictor of diagnosis but also of illness severity and cognitive deficits over time. We found that the SZ PRS predicts a course of persistent negative symptoms. Importantly, among patients initially diagnosed with a psychotic affective disorder, a higher SZ PRS predicted whose diagnosis would change to non-affective psychosis by the 20-year follow-up. The effect requires replication, but accuracy in the highest decile reached a promising 68%.

We tested the association of the SZ PRS with global illness severity and the major symptom domains (hallucinations/delusions, disorganization, avolition, inexpressivity, and depression). Our results suggest that the SZ PRS is closely linked to avolition. Furthermore, the SZ PRS establishes a set degree of severity relative to others with the same illness, rather than different illness trajectories. We found similar associations between the SZ PRS and cognition. The SZ PRS predisposes people with psychotic disorders to consistently worse cognition—specifically, a one standard deviation increase in PRS would predict a 5-point lower IQ—rather than to cognitive decline after illness onset. This is consistent with cognitive deficits observed in high-risk cohorts who have not yet had a psychotic episode^42^, as well as the effect of the cognitive PRS in schizophrenia cohorts^43^. Furthermore, the stability of the SZ PRS’ effects after first admission suggests genetic liabilities for schizophrenia take their toll in the prodromal phase, or before. Taken together, the findings support a neurodevelopmental continuum model of psychosis, such that increased genetic burden predicts cognitive deficits and negative symptoms that emerge prior to eventual diagnosis of non-affective psychosis^44^. Attempts to identify mechanisms through which the SZ PRS exerts its effects should focus on prodromal samples.

Allardyce et al.^3^ found the SZ PRS discriminated psychotic bipolar disorder from schizophrenia. Here, we extend that finding, showing that the SZ PRS discriminates more broadly between affective and non-affective psychosis. These findings also parallel those of Vassos et al.^14^, who show the SZ PRS to be elevated in schizophrenia as compared with other psychotic disorders at first episode. Here, we show that not only is the SZ PRS elevated in schizophrenia in comparison with affective psychosis but also that an elevated SZ PRS predicts an eventual diagnosis of non-affective psychosis. Taken together, these results suggest the core pathology of schizophrenia—cognitive deficits and negative symptoms^18,45^—were present at first admission, but obscured by comorbid mood symptoms. In the months and years following first admission, affective and positive symptoms may subside, while negative and cognitive symptoms persist, resulting in a change of diagnosis to non-affective psychosis. In sum, the precision of the SZ PRS may improve at later stages in the course of illness.

For common disorders such as coronary artery disease, type 2 diabetes, and breast cancer, PRS scores are already showing potential clinical utility^46^. As the size of the Psychiatric Genomic Consortium sample grows, so will the statistical power of the SZ PRS^41^. In psychiatric disorders, the accuracy and therefore utility of PRS scores is challenged by low base rates. However, within samples of psychotic individuals, and especially at the extreme tails of the distribution of scores, the SZ PRS may identify a subset of patients with poor prognoses even when the initial diagnostic picture is unclear.

### Strengths and limitations

The design of this study is unique in that it is the only study that has linked course of illness to the SZ PRS. As such, it was well-positioned to speak to the clinical correlates of genetic liabilities for schizophrenia as they unfold over time. Still, our analyses are limited in three notable ways. First, of the 628 cases who met inclusion criteria only 249 consented to genetic assays at the 20-year follow-up. An analysis of missing data shows few consistent patterns. Those who provided DNA were more likely to be prescribed antipsychotics across the follow-up period, but this trend is difficult to interpret. The prescription may reflect greater symptom severity, or symptoms may be less severe due to being treated, but neither of these trends were reflected in measures of symptom severity. Second, the sample size is modest (249 subjects with psychotic disorders and 205 who were never psychotic). Repeated clinical assessments over time increased the precision of the estimates, but we do not know whether a larger sample would detect effects that were not found here. Finally, predictive modeling always requires cross-validation in a new sample. While our in-sample analyses suggest the effect of the SZ PRS is robust, replication is needed. Importantly, the primarily European ancestry of the Psychiatric Genomic Consortium reduces the predictive power of polygenic risk scores in other ancestry groups^39^. While the SZ PRS is useful in this sample, which is 85% of European ancestry, it will be substantially less so in non-European individuals. In sensitivity analyses completed in a subset of relatively homogenous ancestry, the effects of the SZ PRS on diagnostic shifts from AP to NAP did not reach statistical significance. As the PGC develops PRS scores in other ancestry groups, it will be important to determine whether a similar pattern of results holds.

## Conclusions

The SZ PRS predisposes individuals to consistently worse course of illness severity, avolition, and cognitive deficits. Among those patients with a mix of affective and non-affective symptoms at baseline, high SZ PRS scores predicted those whose diagnosis would shift to non-affective psychosis in the 20 years following first admission. Together, these findings suggest the SZ PRS is an indicator of the core clinical correlates of schizophrenia. They also highlight the potential of the SZ PRS as a diagnostic aid and prognostic indicator for patients with especially high scores, who may benefit from early and consistent monitoring and care.

## Supplementary information

Supplemental Material

Figure S1 source file

## Footnotes

1 The model of cognition at 24 months failed to converge when adjusted for avolition, likely due to the high correlation between the two traits.

2 In this prediction analysis, the effect of mood stabilizers, but not the SZ PRS, survived control of the FDR (SZ PRS *p*-value = 0.04, critical value = 0.03).
