# Supplementary material for: Schizophrenia polygenic risk score and 20-year course of illness in psychotic disorders
Source: Transl Psychiatry. 2019 Nov 14;9:300. doi: 10.1038/s41398-019-0612-5 (PMC6856168; doi:10.1038/s41398-019-0612-5)
Supplement: Supplementary file 1 — Supplemental Material [file 41398_2019_612_MOESM1_ESM.docx]

| **Supplemental Table 1** | | | | | | | | | | | | | | | | | | | |
| --- | --- | --- | --- | --- | --- | --- | --- | --- | --- | --- | --- | --- | --- | --- | --- | --- | --- | --- | --- |
| *Effect of PRS thresholds on diagnostic group means at 20-year follow up* | | | | | | | | | | | | | | | | | | | |
|  | SZ (N=133) | | | BP (N=64) | | | MDD (N=21) | | | | Drug Induced (N=12) | | | Other (N=19) | | | | |  |
|  | R^2^ | *d* | p | R^2^ | *d* | p | R^2^ | *d* | p | R^2^ | | *d* | p | R^2^ | *d* | p | |  |  |
| GWAS | 0.00 | 0.17 | 0.12 | 0.01 | 0.14 | 0.34 | 0.01 | 0.37 | 0.11 | 0.01 | | -0.10 | 0.73 | 0.01 | 0.47 | 0.05 | |  |  |
| p < 0.001 | 0.04 | 0.58 | 0.00 | 0.01 | 0.04 | 0.80 | 0.08 | 0.71 | 0.00 | 0.00 | | 0.26 | 0.39 | 0.01 | 0.66 | 0.04 | |  |  |
| p < 0.01 | 0.06 | 0.60 | 0.00 | 0.02 | 0.06 | 0.56 | 0.05 | 0.55 | 0.02 | 0.01 | | 0.36 | 0.23 | 0.03 | 0.78 | 0.00 | |  |  |
| p < 0.05 | 0.05 | 0.53 | 0.00 | 0.02 | 0.05 | 0.68 | 0.04 | 0.46 | 0.05 | 0.00 | | 0.30 | 0.31 | 0.03 | 0.77 | 0.02 | |  |  |
| p < 0.10 | 0.06 | 0.56 | 0.00 | 0.03 | 0.06 | 0.58 | 0.03 | 0.44 | 0.06 | 0.00 | | 0.29 | 0.34 | 0.03 | 0.81 | 0.02 | |  |  |
| p < 0.20 | 0.06 | 0.54 | 0.00 | 0.03 | 0.05 | 0.68 | 0.03 | 0.46 | 0.05 | 0.00 | | 0.27 | 0.37 | 0.03 | 0.79 | 0.03 | |  |  |
| p < 0.50 | 0.05 | 0.53 | 0.00 | 0.02 | 0.02 | 0.83 | 0.03 | 0.42 | 0.07 | 0.00 | | 0.24 | 0.42 | 0.04 | 0.81 | 0.02 | |  |  |
| *Note.* R^2^ is the coefficient of determination on the liability scale, corrected for case-control ascertainment. PRS scores are normed according to the mean and standard deviation of the never-psychotic group. SZ = schizophrenia and schizoaffective disorder; BP = bipolar disorder; MDD = major depressive disorder. | | | | | | | | | | | | | | | | |  |  |  |

**Supplemental Table 2**

*Analysis of patterns of missing data among cases*

|  | DNA | | | No DNA | | | | |  |  |  |  |
| --- | --- | --- | --- | --- | --- | --- | --- | --- | --- | --- | --- | --- |
| *Categorical Variables* | N | % | N | | | % | Cramer’s V | | | | | |
| Gender |  |  |  | | |  | 0.02 | | | | | |
| Male | 142 | 57.03% | 223 | | | 58.84% |  | | | | | |
| Female | 107 | 42.97% | 156 | | | 41.16% |  | | | | | |
| Ethnicity |  |  |  | | |  | 0.10 | | | | | |
| Caucasian | 202 | 81.12% | 277 | | | 73.09% |  | | | | | |
| African-American | 27 | 10.84% | 65 | | | 17.15% |  | | | | | |
| Other | 20 | 8.03% | 37 | | | 9.76% |  | | | | | |
| BL diagnosis |  |  |  | | |  | 0.02 | | | | | |
| NAP | 152 | 61.04% | 237 | | | 62.53% |  | | | | | |
| AP | 97 | 38.96% | 142 | | | 37.47% |  | | | | | |
| 20Y diagnosis |  |  |  | | |  | 0.00 | | | | | |
| NAP | 164 | 65.86% | 249 | | | 65.70% |  | | | | | |
| AP | 85 | 34.14% | 130 | | | 34.30% |  | | | | | |
| Antipsychotic |  |  |  | | |  |  | | | | | |
| BL |  |  |  | | |  | 0.02 | | | | | |
| Yes | 43 | 17.27% | 71 | | | 18.93% |  | | | | | |
| No | 206 | 82.73% | 304 | | | 81.07% |  | | | | | |
| 6M |  |  |  | | |  | 0.03 | | | | | |
| Yes | 71 | 28.51% | 119 | | | 31.73% |  | | | | | |
| No | 178 | 71.49% | 256 | | | 68.27% |  | | | | | |
| 24M |  |  |  | | |  | 0.14 | | | | | |
| Yes | 94 | 38.06% | 178 | | | 52.35% |  | | | | | |
| No | 153 | 61.94% | 162 | | | 47.65% |  | | | | | |
| 48M |  |  |  | | |  | 0.17* | | | | | |
| Yes | 115 | 46.18% | 235 | | | 63.69% |  | | | | | |
| No | 134 | 53.82% | 134 | | | 36.31% |  | | | | | |
| 10Y |  |  |  | | |  | 0.10* | | | | | |
| Yes | 89 | 38.03% | 95 | | | 47.50% |  | | | | | |
| No | 145 | 61.97% | 105 | | | 52.50% |  | | | | | |
| 20Y |  |  |  | | |  | 0.17* | | | | | |
| Yes | 98 | 40.83% | 44 | | | 60.27% |  | | | | | |
| No | 142 | 59.17% | 29 | | | 39.73% |  | | | | | |
| *Continuous Variables* | N | Mean | N | | Mean | | | | | Cohen’s *d* |  |  |
| Age |  |  |  | |  | | | | |  |  |  |
| BL | 249 | 28.66 | 379 | | | 30.38 | | -0.18* | | | |  |
| 20Y | 249 | 48.18 | 136 | | | 49.22 | | -0.11 | | | |  |
| BL | 249 | 10.95 | 379 | | | 11.77 | | -0.09 | | | |  |
| 6M | 217 | 2.59 | 252 | | | 3.33 | | -0.12 | | | |  |
| SAPS P |  |  |  | | |  | |  | | | |  |
| 24M | 209 | 2.96 | 212 | | | 3.02 | | -0.01 | | | |  |
| 48M | 192 | 2.56 | 165 | | | 3.19 | | -0.11 | | | |  |
| 10Y | 234 | 3.50 | 200 | | | 4.87 | | -0.18 | | | |  |
| 20Y | 246 | 4.39 | 86 | | | 3.49 | | 0.12 | | | |  |
| SAPS D |  |  |  | | |  | |  | | | |  |
| BL | 249 | 7.18 | 379 | | | 6.36 | | 0.13 | | | |  |
| 6M | 217 | 2.23 | 252 | | | 2.06 | | 0.04 | | | |  |
| 24M | 209 | 2.39 | 212 | | | 2.69 | | -0.07 | | | |  |
| 48M | 192 | 2.58 | 158 | | | 2.90 | | -0.07 | | | |  |
| 10Y | 222 | 3.06 | 158 | | | 2.90 | | 0.03 | | | |  |
| 20Y | 246 | 4.77 | 80 | | | 4.04 | | 0.12 | | | |  |
| SANS E |  |  |  | | |  | |  | | | |  |
| BL | 248 | 7.58 | 379 | | | 6.51 | | 0.14 | | | |  |
| 6M | 217 | 6.54 | 252 | | | 7.06 | | -0.06 | | | |  |
| 24M | 209 | 5.78 | 212 | | | 5.89 | | -0.01 | | | |  |
| 48M | 191 | 4.82 | 164 | | | 6.40 | | -0.21 | | | |  |
| 10Y | 219 | 4.58 | 138 | | | 5.86 | | -0.17 | | | |  |
| 20Y | 239 | 7.09 | 23 | | | 10.09 | | -0.32 | | | |  |
| SANS A |  |  |  | | |  | |  | | | |  |
| BL | 248 | 10.16 | 379 | | | 8.89 | | 0.18* | | | |  |
| 6M | 217 | 9.24 | 252 | | | 9.12 | | 0.02 | | | |  |
| 24M | 209 | 8.02 | 212 | | | 8.76 | | -0.10 | | | |  |
| 48M | 192 | 7.54 | 164 | | | 9.20 | | -0.23* | | | |  |
| 10Y | 234 | 9.03 | 201 | | | 10.47 | | -0.16 | | | |  |
| 20Y | 244 | 13.28 | 85 | | | 12.30 | | 0.10 | | | |  |
| GAF |  |  |  | | |  | |  | | | |  |
| BL | 249 | 57.36 | 378 | | | 59.13 | | -0.12 | | | |  |
| 6M | 221 | 56.53 | 269 | | | 56.21 | | *0*.02 | | | |  |
| 24M | 222 | 58.29 | 262 | | | 57.94 | | 0.02 | | | |  |
| 48M | 224 | 59.63 | 241 | | | 58.21 | | 0.09 | | | |  |
| 10Y | 238 | 55.07 | 212 | | | 53.48 | | 0.10 | | | |  |
| 20Y | 239 | 45.63 | 123 | | | 45.94 | | -0.02 | | | |  |
| SCID Dep |  |  |  | | |  | |  | | | |  |
| BL | 249 | 17.54 | 379 | | | 17.35 | | 0.04 | | | |  |
| 6M | 217 | 13.41 | 251 | | | 13.29 | | 0.03 | | | |  |
| 24M | 209 | 12.71 | 210 | | | 12.44 | | 0.06 | | | |  |
| 48M | 192 | 10.67 | 167 | | | 10.62 | | 0.01 | | | |  |
| 10Y | 231 | 11.93 | 186 | | | 11.73 | | 0.05 | | | |  |
| 20Y | 233 | 12.10 | 81 | | | 11.56 | | 0.15 | | | |  |
| *Cognition* |  |  |  | | |  | |  | | | |  |
| VP1 |  |  |  | | |  | |  | | | |  |
| 24M | 191 | 15.97 | 177 | | | 15.96 | | 0.00 | | | |  |
| 20Y | 224 | 14.58 | 12 | | | 14.08 | | *0*.12 | | | |  |
| VP2 |  |  |  | | |  | |  | | | |  |
| 24M | 188 | 6.81 | 175 | | | 6.63 | | 0.12 | | | |  |
| 20Y | 221 | 6.53 | 11 | | | 6.73 | | -0.12 | | | |  |
| VR1 |  |  |  | | |  | |  | | | |  |
| 24M | 200 | 28.23 | 187 | | | 28.10 | | 0.02 | | | |  |
| 20Y | 220 | 25.51 | 12 | | | 24.67 | | 0.11 | | | |  |
| VR2 |  |  |  | | |  | |  | | | |  |
| 24M | 198 | 21.71 | 186 | | | 22.61 | | -0.09 | | | |  |
| 20Y | 216 | 19.24 | 12 | | | 17.58 | | 0.18 | | | |  |
| SD |  |  |  | | |  | |  | | | |  |
| 24M | 193 | 42.98 | 186 | | | 42.25 | | 0.06 | | | |  |
| 20Y | 223 | 39.03 | 12 | | | 34.08 | | 0.43 | | | |  |
| TMT A |  |  |  | | |  | |  | | | |  |
| 24M | 198 | 38.12 | 187 | | | 37.43 | | 0.04 | | | |  |
| 20Y | 215 | 41.08 | 12 | | | 43.92 | | -0.13 | | | |  |
| TMT B |  |  |  | | |  | |  | | | |  |
| 24M | 191 | 92.09 | 182 | | | 91.31 | | 0.02 | | | |  |
| 20Y | 212 | 110.32 | 12 | | | 127.17 | | -0.34 | | | |  |
| COWAT |  |  |  | | |  | |  | | | |  |
| 24M | 194 | 34.21 | 182 | | | 34.14 | | 0.01 | | | |  |
| 20Y | 219 | 35.05 | 12 | | | 26.83 | | *0*.60* | | | |  |
| VOCAB |  |  |  | | |  | |  | | | |  |
| 24M | 201 | 17.99 | 191 | | | 18.12 | | -0.02 | | | |  |
| 20Y | 217 | 19.04 | 13 | | | 18.15 | | 0.15 | | | |  |
| STROOP |  |  |  | | |  | |  | | | |  |
| 24M | 183 | 88.73 | 177 | | | 87.64 | | 0.05 | | | |  |
| 20Y | 185 | 86.19 | 12 | | | 72.08 | | 0.58 | | | |  |

*Note.* Contrasts are between those who provided DNA versus those who did not. BL = Baseline; NAP = Non-Affective Psychosis; AP = Affective Psychosis; AP Medication = Antipsychotic Medication; VP1 = Verbal Paired Associates, Immediate Recall; VP2 = Verbal Paired Associates, Delayed Recall; VR1 = Visual Reconstruction, Immediate Recall; VR2 = Visual Reconstruction, Delayed Recall; SD = Symbol-Digit Modalities; TMT A = Trail Making Test, part A; TMT B = Trail-Making Test, part B; COWAT = Controlled Oral Word Association Task; Vocab. = Vocabulary; * p < 0.05.

| **Supplemental Table 3** | |  | | |  |  | |  | | |  |  |
| --- | --- | --- | --- | --- | --- | --- | --- | --- | --- | --- | --- | --- |
| *PRS scores by diagnostic group* | |  | | |  |  | |  | | |  |  |
|  | | | Baseline | | |  | | | 20 years | | |  |
| Diagnosis | N | | R^2^ | *d* | | p | N | | R^2^ | *d* | | p |
| Schizophrenia/Schizoaffective | 73 | | 0.04 | 0.56 | | <0.01 | 133 | | 0.06 | 0.68 | | <0.01 |
| Other Psychoses | 67 | | 0.06 | 0.63 | | <0.01 | 19 | | 0.03 | 0.55 | | 0.07 |
| Substance-Induced Psychosis | 12 | | 0.10 | 0.56 | | <0.01 | 12 | | 0.01 | 0.23 | | 0.40 |
| Bipolar Disorder | 62 | | 0.03 | 0.49 | | <0.01 | 64 | | 0.02 | 0.37 | | 0.01 |
| Major Depression | 35 | | 0.03 | 0.43 | | 0.01 | 21 | | 0.05 | 0.58 | | 0.02 |
| *Note*. R^2^ is Lee’s (2012) coefficient of determination on the liability scale, corrected for case-control ascertainment. Cohen’s *d* is calculated relative to never-psychotic adults. | | | | | | | | | | | |  |

| **Supplemental Table 4** | | | | | | | | | | | | |
| --- | --- | --- | --- | --- | --- | --- | --- | --- | --- | --- | --- | --- |
| *Symptom trajectories over 20 years, Caucasian participants* | | | | | | | | | | | | |
|  | Mean Effect of SZ PRS | | | | SZ PRS on Course Part 1 | | | | SZ PRS on Course Part 2 | | | |
|  | R^2^ | β | 95% CI | p | R^2^ | β | 95% CI | p | R^2^ | β | 95% CI | p |
| Hallucinations/Delusions | <0.01 | .02 | [-.16, .20] | 0.83 | <0.01 | -.05 | [-.19, .10] | 0.54 | - | - | - | - |
| Disorganization | 0.01 | .08 | [-.11, .27] | 0.39 | <0.01 | .02 | [-.14, .17] | 0.82 | <0.01 | .03 | [-.14, .20] | 0.70 |
| Avolition | **0.05** | **.22** | **[.02, .42]** | **0.03** | 0.01 | -.07 | [-.17, .04] | 0.24 | <0.01 | .02 | [-.09, .14] | 0.69 |
| Inexpressivity | 0.02 | .14 | [-.06, .35] | 0.18 | 0.01 | -.11 | [-.23, .01] | 0.08 | <0.01 | -.03 | [-.16, .10] | 0.64 |
| Depression | 0.02 | .15 | [-.02, .32] | 0.08 | **0.02** | **-.13** | **[-.01, -.25]** | **0.04** | - | - | - | - |
| Illness severity | **0.07** | **-.26** | **[-.47, -.05]** | **0.02** | <0.01 | -.01 | [-.12, .09] | 0.81 | <0.01 | .04 | [-.06, .13] | 0.45 |
| *Note*. Results are based on N=235 cases within three standard deviations of the mean on the first four principal components of population stratification. The mean effect of the SZ PRS indicates an effect that is constant across all time points. SZ PRS on course is the effect of the SZ PRS on change in symptoms over time. For Reality Distortion and Depression, symptom trajectories are linear, so there is only one effect on slope. Trajectories have inflection points for Disorganization (13.5 years after baseline), Avolition (4.5 years), Inexpressivity (2.5 years), and Illness severity (4.5 years), so effect of PRS is also modeled on the second slope for these domains. | | | | | | | | | | | | |

| **Supplemental Table 5** | | | | | | | |
| --- | --- | --- | --- | --- | --- | --- | --- |
| *Predictive accuracy of SZ PRS* | | | | | | | |
| Decile | Sensitivity | PPV | Specificity | NPV | Accuracy |  |  |
| First | 0.97 | 0.34 | 0.14 | 0.90 | 0.40 | |  |
| Second | 0.84 | 0.34 | 0.23 | 0.75 | 0.42 | |  |
| Third | 0.81 | 0.37 | 0.35 | 0.79 | 0.49 | |  |
| Fourth | 0.74 | 0.40 | 0.47 | 0.79 | 0.56 | |  |
| Fifth | 0.65 | 0.41 | 0.56 | 0.77 | 0.59 | |  |
| Sixth | 0.48 | 0.38 | 0.64 | 0.72 | 0.59 | |  |
| Seventh | 0.35 | 0.38 | 0.73 | 0.71 | 0.61 | |  |
| Eight | 0.26 | 0.40 | 0.82 | 0.70 | 0.64 | |  |
| Ninth | 0.16 | 0.50 | 0.92 | 0.70 | 0.68 | |  |
| *Note.* Deciles reflect distribution of SZ PRS among those presenting with affective psychosis at admission. A “positive” is an individual who is diagnosed with an affective psychosis at baseline, but who, at 20 year follow-up, has shifted into the non-affective psychosis group. A “negative” is an individual with stable affective psychosis. PPV = positive predictive value; NPV = negative predictive value. | | | | | | | |

**Sensitivity Analyses**

*Substance Induced Psychosis*

When participants with a diagnosis of substance-induced psychosis at either baseline or the 20-year follow-up were removed from the analysis, the magnitude of the effects of PRS on diagnostic shifts remained the same (stable NAP versus stable AP *d*=0.31, p<0.05; stable NAP versus shift into NAP *d*=0.10, p=0.82), but the contrast between stable AP and shift into NAP groups was no longer statistically significant (*d*=0.41, p=0.06).

*Other Psychosis*

When participants with other psychotic disorders at either baseline or 20-year follow-up were removed from the analysis, the contrast between stable NAP versus stable AP *d*=0.25, p=0.13 was no longer significant, but the magnitude of the effects of PRS on diagnostic shifts remained approximately the same (stable NAP versus shift into NAP *d*=0.26, p=0.82, stable AP and shift into NAP *d*=0.54, p=0.01).

*Population stratification*

Results of SZ PRS on symptom trajectories were the same in the subsample of cases within three standard deviations of the mean on the first four principal components of population stratification (N = 235), with the addition of an effect of SZ PRS on depression change over the course of illness, such that those with higher SZ PRSs tended to have fewer depression symptoms over the illness course (R^2^=0.02; β=-0.13, [-0.25, -0.01], p = 0.04). SZ PRS was associated with poorer cognition at 24 months (R^2^=0.06; β=-0.24, [-0.44, -0.05], p = 0.02) and at 20 years (R^2^=0.07; β=-0.27, [-0.45, -0.08], p < 0.01). Contrasts between diagnostic shift groups were no longer significant (stable AP versus stable NAP *d* = 0.22, p = 0.18; stable AP versus shift into NAP *d =* 0.43, p = 0.07), but effect sizes were of a similar magnitude of those observed in the full sample.

**Figure Legend**

Figure S1

*Note.* A “positive” is an individual who is diagnosed with an affective psychosis at baseline, but who, at 20 year follow-up, has shifted into the non-affective psychosis group. Distance to the point of perfect classification (0,1) is minimized at the 68.5%ile of scores observed among never-psychotic adults. A “negative” is an individual with stable affective psychosis. FPR = false positive rate; TPR = true positive rate.
