# Supplementary figures and images for: Schizophrenia polygenic risk score and 20-year course of illness in psychotic disorders
Source: Transl Psychiatry. 2019 Nov 14;9:300. doi: 10.1038/s41398-019-0612-5 (PMC6856168; doi:10.1038/s41398-019-0612-5)

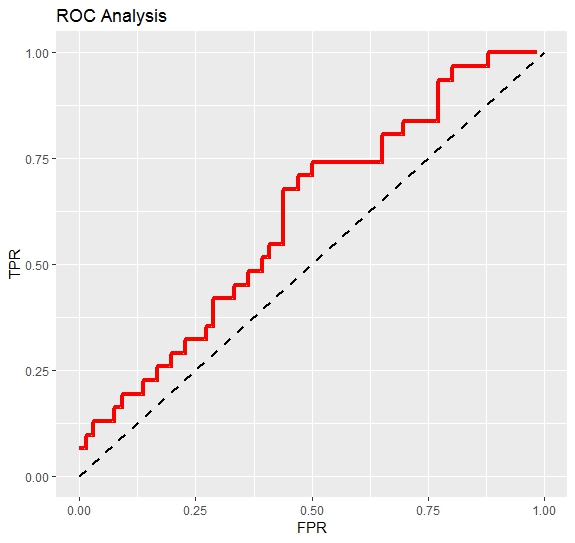

Supplement: Supplementary file 2 — Figure S1 source file [file 41398_2019_612_MOESM2_ESM.jpg]
